# A kind mind: effects of compassion-based meditation on prosocial intergroup outcomes in a South African pilot sample

**DOI:** 10.3389/fpsyg.2025.1450549

**Published:** 2025-07-11

**Authors:** Adala M. Prevost, Michal George, Melike M. Fourie

**Affiliations:** ^1^Department of Psychology, Stellenbosch University, Stellenbosch, South Africa; ^2^Institute for Mindfulness South Africa, Stellenbosch University, Stellenbosch, South Africa; ^3^Department of Surgery, Neuroscience Institute, University of Cape Town, Cape Town, South Africa

**Keywords:** compassion, meditation, self-transcendence, prejudice, prosocial, intergroup

## Abstract

Three decades into democracy, the corollaries of apartheid continue to pattern South African society, with complicated race feelings and resistance to reparative government policies still driving separation. Sharing a grounding with African knowledge systems in the interconnectedness of all people, compassion-based meditation has proven to be a powerful promoter of prosocial action toward strangers and stigmatized groups abroad. It is, however, unclear whether such findings would translate to South Africa with its history of racialised conflict. Here, we piloted a mixed methods study to examine whether 8 weeks of compassion-based meditation would foster positive intergroup attitudes and prosocial outcomes, beyond personal wellbeing, in a White South African sample. We found greater compassion self-practice to be associated not only with significantly increased life satisfaction and reduced stress, but also with heightened outgroup compassion and reduced desire for social distance. Furthermore, post intervention, we observed significantly reduced racial prejudice, increased intergroup contact, and greater prosocial outcomes expressed in support toward collective action and restitutive government policies. Qualitatively, participants reported broadened compassion and affiliation with strangers, suggesting enhanced self-transcendence. These findings offer early support for the potential of compassion-based meditation to improve intergroup relations locally. Future directions include a randomized controlled trial in an appropriately powered sample, and expansion of the methodology to include other social groups.

## Introduction

During apartheid South Africa, racialised categories^1^ of White, Indian, Colored, and Black African were utilized as iconic signifiers of social rank, enforcing limits to people's humanity (Posel, 2001). These race labels persist in contemporary South Africa, trailing along the unresolved pain, division, and violence of their histories, despite significant efforts to redress the power differential (Fourie et al., 2022; Hammett, 2010). While the fall of apartheid undoubtedly ushered in a new era for South Africans, stubborn political divides between racialized groups often dictate the behavior of voters during general elections. Notably, opposition to post-apartheid restorative policies suggests that lingering prejudicial feelings may continue to operate covertly (Dixon et al., 2010a; Fourie and Moore-Berg, 2022; Fourie et al., 2017).

Social identity theory holds that belonging to a social group is associated with the harboring of more positive appraisals of ingroups vs. outgroups, affording a self-esteem gain (Tajfel et al., 1971). Favorable contact with outgroup members, as proposed by Allport (1954), is one of the most well-evidenced strategies to aid in reversing this process through humanizing the “other”^2^ (Pettigrew and Tropp, 2006). In situations of high prejudice, however, Allport's optimal conditions for contact are not always easily met (Holtman et al., 2005; Pettigrew, 1998), with negative intergroup contact risking a prejudice surge (Barlow et al., 2012). In the South African context, Dixon et al. (2007, 2010b) reported contact-related reductions in prejudice in both Black and White groups, however, those gains did not necessarily predict meaningful prosocial outcomes. While positive intergroup contact undeniably contributes to prejudice reduction and prosocial outcomes amongst advantaged members (Hässler et al., 2020, 2021; Turner et al., 2020), it is not a panacea for stimulating reparative action in historically divided societies (McKeown and Dixon, 2017).

Much intergroup research emphasizes the key role of empathy as a driver of prosocial motivation for outgroups (Finlay and Stephan, 2000; Mealy and Stephan, 2010). Empathy is a multifaceted emotional process, however, with affective sharing, perspective taking, and empathic concern associated with different subjective and behavioral outcomes (Decety and Cowell, 2015). Perspective taking, or the ability to mentally place oneself in another's situation, has been associated with lower levels of prejudice against African Americans in White American college students (Dovidio et al., 2004). Similarly, Vorauer and Sasaki (2009) reported a reduction in prejudice in White Canadians when asked to adopt an imagine-other perspective when watching a documentary showing the adversities of Aboriginal Canadians. However, this gain disappeared when participants had direct contact with outgroup members and was even reversed in high-prejudice individuals.

Empathic concern, which involves other-oriented feelings of compassion or sympathy for someone suffering, is typically associated with altruistic motivation and care-giving behaviors (Batson et al., 2015). Yet various personal and situational factors influence whether and toward whom empathic concern is extended (Zaki, 2014). Empathy-based interventions, for example, may unintentionally foster disproportionate ingroup empathy, which has been associated with increased outgroup harm and reduced helping behavior (Bruneau et al., 2017). In addition, in contexts where (historically) advantaged group members are benefited by the status quo, feelings of concern for those marginalized might not produce prosocial change, but rather serve to mask continued structural inequality (Fourie and Verwoerd, 2022; Dixon et al., 2010a). Cialdini et al. (1997) proposed that for empathic concern to lead to prosocial outcomes, an integration of the other into the self-concept is needed. In line with this view, Decety and Cowell (2015) postulated that the lower levels of prosocial mobilization they observed in individuals with a high social status was linked to a reduced incorporation of outgroup members in their self-construals. How self-other affiliation is mentally constructed may therefore act to modulate prosocial outcomes.

Selfhood is inexorably tied to group identity in African philosophical frameworks, which are ontologically rooted in the premise that the individual derives wholeness from communal belonging (Ramose, 1999). This is clearly expressed in the principle of *Ubuntu*, articulated by Tutu (1999, p. 31) as: “My humanity is caught up, is inextricably bound up, in yours.” Accordingly, African models of social healing conceptualize personal wellbeing as inseparable from that of the community, framing reconciliation in the aftermath of conflict as arising out of mutual recognition, empathy, and social reintegration (Tutu, 1999). This worldview has guided processes such as South Africa's Truth and Reconciliation Commission, where social repair was enacted through truth-telling, public acknowledgment of harm, and collective forgiveness (Murithi, 2006).

Indigenous social healing traditions from other contexts include the Hawaiian practice of *Ho'oponopono*, where a mutual release from grievance is achieved through confession, remorse, and the requesting of forgiveness (Yates, 2014). Translating to reciprocity in Quechua, the Andean philosophy of *Ayni* emphasizes cooperation between people, as well as with a living earth (*Pachamama*) and the ancestors, as fundamental to a healthy society (Apffel-Marglin, 2011). The Navajo justice and harmony ceremony of *Hozhooji Naat‘aanii* negotiates peacemaking via a horizontal system where all views and emotional experiences are considered equal in the restoration of solidarity (Bluehouse and Zion, 1993).

Deriving from Eastern contemplative traditions and a relative newcomer to Western scientific research, compassion-based meditation (CBM) acts on the limits of empathy. The CBM practices of mindfulness, loving-kindness, and compassion meditation are seen to cultivate *mettā*, translating to a love that is universal, pure, benevolent, and unattached (Gilbert, 2005; Hutcherson et al., 2008; Leaviss and Uttley, 2014). The new millennium has seen the rise of CBM practices in the West, popularized for their ability to boost happiness and foster a sense of connectedness, even with those outside of relational circles (Condon et al., 2013; Galante et al., 2014; Hofmann et al., 2011; Hutcherson et al., 2008; Kang et al., 2014; Weng et al., 2013). Strikingly, increases in positive regard, affiliation, and altruistic behavior toward neutral strangers have been observed even after a single session of CBM (Hutcherson et al., 2008; Leiberg et al., 2011; Weng et al., 2013), and also when faced with their suffering (Klimecki et al., 2012).

Underpinned by the affiliative neurochemical systems and shown to activate feelings of interpersonal connection and trust, CBM has been found to lower threat and stress responses and is associated with a shift in motivation from self-orientation to relational care (Condon et al., 2013; Gilbert, 2019; Kang, 2019). Direct effects reported in this regard include heightened positive emotions, improved emotional regulation, broadened attention, and increased empathy and tolerance for self and others, with accompanying reductions in criticism, judgement, blame and defensiveness (Hofmann et al., 2011; Kang, 2019; Weng et al., 2013). Based on these powerful prosocial effects, recent research has been harnessing CBM in a drive to mitigate abiding intergroup prejudice. Specifically, it has proven effective in reducing implicit (Kang et al., 2014; Stell and Farsides, 2016) and explicit (Hunsinger et al., 2012) bias toward stigmatized groups, as well as raising interest in future contact with outgroups (Parks et al., 2014).

Kang (2019) has proposed that CBM's mechanism of action could be that of self-transcendence, defined here as the progression from a defensive favoring of the self to an attitude of beneficence toward others (Kitson et al., 2020). Self-transcendence in its ultimate form can be seen to extend further than mere empathic concern or a broadening of self-construals, but rather a remapping of self and “other” in a newly forged compassionate and global identity. Manuel (2015) refers to a “natural identity,” a oneness that does not deny difference, but is inclusive of multiplicity. In this way, CBM differs from empathy-based interventions, which can inspire parochialism. Moreover, the self-transcendent state mirrors the constituting of the self-in-the-collective in *Ubuntu's* relational ontology. Being able to experience the “other” as an extension of oneself enables the non-dualistic holding of different viewpoints and a harnessing of common goals (Gilbert, 2019). Further intersecting with *Ubuntu* values, Manuel (2015) describes compassion as a radical act of interconnectedness that recognizes suffering as collective rather than individual.

Taken together, the intrapersonal dissolution of self-other boundaries and boost in positive feelings implicated in CBM may open a pathway to mediate stubborn intergroup divides in the South African post-apartheid setting, where structural inequalities persist and deep psychosocial divides continue to be reinforced by racialised group identities. An intervention that acts on the “otherness” boundary without the limitations of interpersonal approaches, such as contact, may present the long-sought agent of change, where high levels of prejudice and/or defensive self-interest have consistently posed roadblocks to reconciliation. CBM may furthermore be well suited to the historically fractured South African society, as it operationalises the *Ubuntu* ethic through realigning personal with collective orientation. With principles and objectives auspiciously congruent with those of African social healing paradigms, CBM may uniquely contribute to recognition and transforming of racialised suffering in societies with intergenerational trauma (Manuel, 2015).

Here we examined whether a CBM intervention can reduce intergroup prejudice and enhance prosocial behavior, potentially through the mechanism of self-transcendence, in a local setting using a mixed methods approach. We recruited a pilot sample of White South African adults to explore the effects of 8 weeks of CBM practice on self-reported wellbeing, prejudice, and other intergroup outcomes. Although this intervention can realistically be expected to hold benefits for all social groups, we elected to pilot the study in a White demographic, to which the authors correspond.^3^ This decision was motivated by the goal of encouraging meaningful, reparative social action in the social group favored under apartheid.

We hypothesized that following the CBM intervention, (i) *subjective wellbeing* would increase significantly (Whitesman et al., 2018), (ii) *racial prejudice* would decrease significantly (Hunsinger et al., 2012), (iii) *intergroup contact and compassion* would increase significantly (Parks et al., 2014), and that (iv) *prosocial intergroup outcomes* would improve significantly (Zheng et al., 2023). We further argued that changes in these measures would be significantly associated with CBM self-practice. Finally, we analyzed participants' open-ended responses to assess whether these converged with the quantitative results, and to gain a richer understanding of their experiences.

## Methods

### Participants

We recruited White participants through online platforms and community notice boards. Exclusion criteria included an ongoing or past contemplative practice exceeding one weekly session, with contemplative practice defined as incorporating a single point of focus, such as mindfulness, conscious breathing, Tai Chi, or Qigong (Bruce et al., 2018). In addition, candidates taking recreational drugs or psychoactive medication were excluded to avoid confounding neurochemical influences. Thirty-one eligible participants signed up for the study, of whom 16 completed all study procedures (*M*_*age*_= 40.63, *SD*_*age*_= 14.00; 11 female) and received ZAR300 for their participation. Despite the small sample, our *post-hoc* power analysis indicated that we achieved β > 0.80 for most outcome effects (G^*^Power 3.1; Faul et al., 2007).

### Materials

Subjective wellbeing and intergroup measures were administered online 1 week prior to the CBM intervention (pre-test), and 1 week afterwards (post-test), with items shuffled to counteract expectancy effects. Survey sections are provided in the Supplementary material. Participants were informed that survey items focused on the three largest racialized groups in South Africa, but that we do not endorse the legitimacy of these artificial racial categories.

#### Subjective wellbeing

The Satisfaction with Life Scale (SWLS; Diener et al., 1985) assessed participants' levels of subjective fulfillment (e.g., “In most ways my life is close to my ideal”; 1=*strongly disagree*, 7=*strongly agree*; αs > 0.84), whereas the Perceived Stress Scale (PSS; Cohen et al., 1983) measured subjective global stress (e.g., “In the last 2 weeks, how often have you been angered because of things that were outside of your control?;” 0 = *never*, 4 = *very often*; αs > 0.86).

#### Racial prejudice

Three feeling thermometers assessed participants' affective prejudice toward White, Black African, and Colored groups on a spectrum from cold to warm (0 = *very cold*, 100 = *very warm*; Haddock et al., 1993). Scores were reverse-coded so that higher scores indicate greater prejudice. A South African adaptation of Social Distance Scale (Bogardus, 1925) was used to evaluate affective desire for social distance with racial outgroups. Participants indicated their level of discomfort with three race scenarios (e.g., “It would bother me if my son or daughter ended up marrying a [outgroup] person;” 0 = *strongly disagree*, 100 = *strongly agree*; 0.79 < αs <0.86).

#### Intergroup contact

Four questions assessed the quantity (“In general, how often do you interact?;” 1 = *never*, 7 = *all the time*) and quality (“When you do interact, how often are the interactions positive/pleasant vs. negative/unpleasant?;” 1 = *always negative*, 7 = *always positive*) of interactions with Black African and Colored outgroups (Bruneau et al., 2020).

#### Outgroup compassion

Nine questions, adapted from Bruneau et al. (2015), assessed compassion toward White, Black African, and Colored race groups in scenarios involving adversity (e.g., “Colored people who struggle to make ends meet;” 0 = *not sorry at all*, 100 = *very sorry* (0.79 < αs <0.96).

#### Prosocial intergroup outcomes

These were measured across two scales. Collective action support gauged participants' support for disadvantaged groups engaged in collective action for social repair over four local issues (e.g., “Communities from informal settlements protesting over slow land reform;” 0 = *strongly oppose*, 100 = *strongly support*; 0.78 <αs; Fourie et al., 2022). While these items did not specifically refer to race groups, the issues raised affect those marginalized by apartheid. Petition support assessed participants' willingness to support progressive outgroup policies (Bruneau et al., 2015). Specifically, they indicated whether they would add their signatures to five petitions that support redistributive policies, e.g., affirmative action (1 = *support this petition*,−1=*oppose this petition*, 0=*neither support nor oppose this petition*; αs > 0.72).

#### CBM practice

Following Kang et al. (2014), the post-test additionally asked participants to record (i) the number of workshop sessions they attended, (ii) the number and (iii) total duration of CBM self-practice sessions they conducted over the intervention period, and (iv) their enjoyment of other participants in the group.

#### Open-ended questions

The post-test also asked participants to respond to the following three questions in their own words: (i) “Please share any experiences that stand out for you of the workshop, positive or negative,” (ii) “Please share something important that you learned and what value it had for you with regard to your interactions with other people,” and (iii) “Do you have suggestions for what could have made your experience more rewarding?”.

### Procedure

An online CBM workshop intervention comprising 8 weekly 1-h sessions was hosted on Zoom in this within-group pilot study. Facilitated by an experienced and qualified meditation teacher, these sessions involved an anchoring mindfulness meditation practice, followed by guided loving-kindness and compassion meditation group practice, time for discussion, and guidance on self-practice. The compassion practice was gradually expanded each week, inviting participants to cumulatively include in their visualizations: 1) Someone for whom they have unconditional positive regard; 2) themselves; 3) a family member or friend; 4) other course participants; 5) neutral others in their life; 6) someone with whom they have a difficult relationship; 7) other people in the city/country; and 8) unknown others on the continent and in the world. Participants were also asked to undertake daily 10–15 min CBM self-practice sessions, guided by audio files uploaded for them weekly (see Supplementary material).

### Data analysis

To test our hypotheses, we first analyzed changes in all intergroup outcome variables with paired-samples *t*-tests, controlling for multiple comparisons using the Benjamini-Hochberg procedure and a false discovery rate (FDR) of 10% (Benjamini and Hochberg, 1995). We then examined associations between change scores and intervention-related measures (workshop attendance, number and duration of self-practice sessions, and enjoyment of other participants) with zero-order correlations. Because our hypotheses were directional, these analyses were 1-tailed to achieve greater power. Given the small sample, we inspected 95% confidence intervals (CIs) derived through bootstrapping running 1,000 iterations to help validate our findings (Efron, 1987), and report only associations that survived a Benjamini-Hochberg false discovery rate (FDR) of 10%.

Participants' qualitative reflections on their experiences were explored via thematic analysis (Braun and Clarke, 2006). These analyses were approached inductively, to explore themes emerging for participants, as well as deductively, to examine a priori-determined themes of personal wellbeing and compassion toward others.

## Results

### Quantitative analysis

Descriptive statistics for the test variables are reported in Table 1. As anticipated, paired-samples *t*-tests showed a significant improvement in participants' satisfaction with life [SWLS: *t*(15) = −3.66, *p* < 0.001, Hedges' *g* = −0.87], along with a significant reduction in their perceived stress [PSS: *t*(15) = 4.44, *p* < 0.001, *g* = 1.05], offering support for increased *subjective wellbeing*.

**Table 1:** Descriptive statistics for the study test variables.

**Measures**	**Pre-test**		**Post-test**		**Comparison**	**Effect size**
	***M***	***SD***	***M***	***SD***	**of means (** ***p*** **)**	**Hedges'** ***g***
Satisfaction with life^a^	20.56	7.53	25.69	6.16	**<0.001**	0.87
Perceived stress^b^	20.31	7.99	12.00	5.42	**<0.001**	1.05
Affective prejudice (Σ outgroups)^c^	31.97	13.83	22.66	14.96	**0.003**	0.75
Black African	31.13	14.99	23.88	15.63	**0.016**	0.56
Colored	32.81	14.59	21.44	15.28	**0.001**	0.85
White	29.31	16.89	19.25	13.29	**0.009**	0.63
Social distance (Σ outgroups)^c^	24.67	24.32	20.53	21.85	0.179	0.23
Black African	25.07	24.26	21.33	23.05	0.217	0.20
Colored	24.20	27.11	19.80	21.27	0.196	0.22
Intergroup Contact (Σ outgroups)^d^	4.41	1.05	4.97	0.98	**0.006**	0.70
Black African	4.47	1.12	4.99	0.97	**0.011**	0.63
Colored	4.35	1.11	4.95	1.19	**0.014**	0.60
Compassion (Σ outgroups)^c^	81.13	15.26	84.67	11.41	0.106	0.32
Black African	79.73	15.30	83.27	11.98	0.149	0.26
Colored	82.33	16.77	86.13	11.62	0.081	0.36
Collective action support^c^	67.13	17.79	78.00	13.65	**0.009**	0.64
Petition support^e^	0.04	0.46	0.20	0.52	**0.048**	0.44

Higher scores indicate greater values on all variables. Paired samples t-tests were used to compare means, with significant comparisons surviving the Benjamini-Hochberg threshold for a 10% FDR in bold.

a Scores range from 5 (low) to 35 (high).

b Scores range from 0 (low) to 40 (high).

c Percentage scores.

d Scores range from 1 (low) to 7 (high).

e Scores range from −1 (opposing petition) to 1 (supporting petition).

Regarding measures of *racial prejudice*, we observed significantly reduced affective prejudice on all feeling thermometers [Black African: *t*(15) = 2.36, *p* = 0.016, *g* = 0.56; Colored: *t*(15) = 3.57, *p* = 0.001, *g* = 0.85; White: *t*(15) = 2.65, *p* = 0.009, *g* = 0.63]. Social distance also decreased for both racial outgroups, suggesting a lowering of interracial barriers. These analyses did not reach significance, however (*p*s <0.217; *g*s > 0.20).

In line with expectations, *intergroup contact* following the intervention increased significantly across both racial outgroups [Black African: *t*(14) = −2.59, *p* = 0.011, *g* = 0.63; Colored: *t*(14) = −2.44, *p* = 0.014, *g* = 0.60]. *Compassion* scores for racial outgroups were also higher following the intervention, however these gains were not significant (*p*s <0.149, *g*s > 0.26).

*Prosocial intergroup outcomes* were also more favorable following the intervention: support for collective action showed a significant increase, *t*(15) = −2.68, *p* = 0.009, *g* = 0.64, and participants reported a significantly increased willingness to support petitions that would positively impact outgroup members (historically marginalized groups), *t*(14) = −1.78, *p* = 0.048, *g* = 0.44.

To examine associations between changes in test variables (i.e., post-test score – pre-test score) and CBM practice, we performed zero-order correlations (see Table 2). Higher workshop attendance was significantly associated with more CBM self-practice sessions, *r* = 0.65, *p* = 0.003, and with a longer duration of self-practice, *r* = 0.69, *p* = 0.002, over the 8-week period.

**Table 2:** Descriptive statistics and intercorrelations for CBM practice variables.

**Measures**	**1**.	**2**.	**3**.	**4**.
**CBM Practice Variables**				
1. Workshop attendance^a^	-			
2. CBM self-practice^b^	0.65^**^[0.09, 0.91]	**-**		
3. CBM duration^c^	0.69^**^[0.18, 0.91]	0.96^***^[.82, 0.99]	-	
4. Enjoyment of participants^d^	0.17 [−0.37, 0.64]	0.51^*^[0.02, 0.81]	0.40 [−0.10, 0.75]	–
**Test Variables**				
5. Δ Satisfaction with life	0.24 [−0.38, 0.75]	0.65^**^[0.20, 0.91]	0.59^**^[0.08, 0.90]	0.01 [−0.56, 0.54]
6. Δ Perceived stress	−0.23 [−0.74, 0.41]	−0.50^*^[−0.86, **−0.01]**	−0.48 [−0.84, 0.04]	−0.18 [−0.72, 0.46]
7. Δ Outgroup prejudice	−0.13 [−0.65, 0.43]	−0.22 [−0.84.47]	−0.09 [−0.70, 0.56]	0.12 [−0.46, 0.67]
8. Δ Social distance	−0.55^*^[−0.93, **−0.01]**	−0.46 [−0.87, 0.17]	−0.42 [−0.86, 0.27]	−0.03 [−0.68, 0.66]
9. Δ Intergroup contact	−0.06 [−0.70, 0.54]	0.16 [−0.41, 0.64]	0.18 [−0.35, 0.67]	−0.39 [−0.82, 0.14]
10. Δ Outgroup compassion	0.25 [−0.19, 0.74]	0.60^**^[0.08, 0.87]	0.28 [−0.36, 80]	0.36 [−0.04, 0.69]
11. Δ Collective action	0.06 [−0.56, 0.70]	0.18 [−0.42, 0.74]	0.08 [−0.51, 0.67]	0.39 [−0.24, 0.80]
12. Δ Petition support	−0.03 [−0.52, 0.49]	0.02 [−0.50, 0.55]	0.08 [−0.48, 0.60]	−0.05 [−0.58, 0.60]
*M*	5.94	38.25	8.69	73.06
*SD*	2.08	30.96	5.99	19.38

Spearman's correlations were conducted with 95% bootstrap confidence intervals (CI) in brackets. Significant correlations surviving the Benjamini-Hochberg threshold for a 10% FDR are in bold. CBM, compassion-based meditation.

a Scores range from 1 to 8.

b Total 10-min self-practice sessions over 8 weeks.

c Total duration of self-practice sessions over 8 weeks (in h).

d Enjoyments of other participants in the group, from 1 (low) to 100 (high).

* *p* < 0.05.

** *p* < 0.01.

*** *p* < 0.001.

As predicted, positive changes in satisfaction with life and perceived stress were significantly associated with the number (SWLS: *r* = 0.65, *p* = 0.004; PSS: *r* = −0.50, *p* = 0.028) and duration (SWLS: *r* = 0.59, *p* = 0.010) of CBM self-practice sessions. Furthermore, positive changes in compassion toward racial outgroups were significantly associated with more CBM self-practice sessions, *r* = 0.60, *p* = 0.009. Finally, reduced outgroup social distance was significantly associated with greater workshop attendance, *r* = −0.55, *p* = 0.016. No other intergroup outcomes were significantly associated with CBM practice measures, and notably, none were associated with enjoyment of other participants in the group (*ps* = n.s.). The latter finding suggests that positive intergroup outcomes were not linked to enjoyment of other participants, offering further support for the specific role of CBM practice as the agent of change.

In summary, the more participants engaged in CBM self-practice over the 8-week period, the greater subjective wellbeing they reported, together with greater outgroup compassion and reduced need for social distance. Although positive changes on other intergroup outcomes were also associated with increased CBM self-practice, these correlations did not reach significance after employing the Benjamini-Hochberg FDR threshold.

### Qualitative findings

Central themes were extracted from participants' reflections to support and elucidate the quantitative findings. These were *improved personal wellbeing*, with subthemes of (i) positive effects and (ii) reductions in negative experience; *prosocial outcomes*, with subthemes of (i) improved interpersonal relations, and (ii) increased affiliation; and *mettā*, with (i) broadened compassion and (ii) self-transcendence subthemes.

#### Improved personal wellbeing

Many positive effects of CBM were reported, with participant #7 expressing: “I was able to be more positive, … engaged with people, present, focused, and in tune with my own emotions both positive and negative,” while finding the practice “incredibly calming, relaxing and beneficial to my day.” Participant #11 said: “I was surprised that I was feeling calmer almost with immediate effect.” Reductions in negative experience were also reported. Participant #9 professed to be “less impatient whilst driving,” while participant #3 found the practice a “very powerful tool for reducing irritability,” and participant #7 echoing: “I am less bothered by small irritants in my daily life than I used to be.” Participant #6 reported “reprieve from racing thoughts,” while participant #4 “learned to take a step back, be less reactive and more aware.”

#### Prosocial outcomes

Several participants noticed positive changes in themselves that translated into improved social interactions, with participant #7 finding “being happy in myself leading to being happy, kind, encouraging toward others,” adding that “this transformation in myself led to me being more interested in other people.” Participant #4 expressed that: “Compassionate responses create peaceful and happy situations,” and participant #5 found: “The calmer I am, the better space I am to see the good in others.” Increased affiliation with others was also reported, with participant #7 finding: “I am faster to greet strangers and engage in a conversation.” Participant #6 stated: “I believe that in a country such as South Africa, learning to have a greater sense of others” wellbeing is important, as we have become so desensitized to the poverty and horrible living conditions many in this country face.'

#### Mettã

Most participants reported the desired outcome of the CBM practice, namely, a cultivation of compassion, with participant #12 reporting being “surprised to see how I can easily expand my compassion broadly.” Participant #14 described: “I started looking at people and their actions with more understanding and was also kinder to myself when usually I would be quite harsh.” Participant #10 expressed that the practice “helped me to value those people around me,” while “approaching others and situations and myself with curiosity rather than judgement” was experienced by participant #8. Participant #1 described that the practice helped them: “to have compassion even though you do not agree or understand someone's actions.”

**Figure 1:**
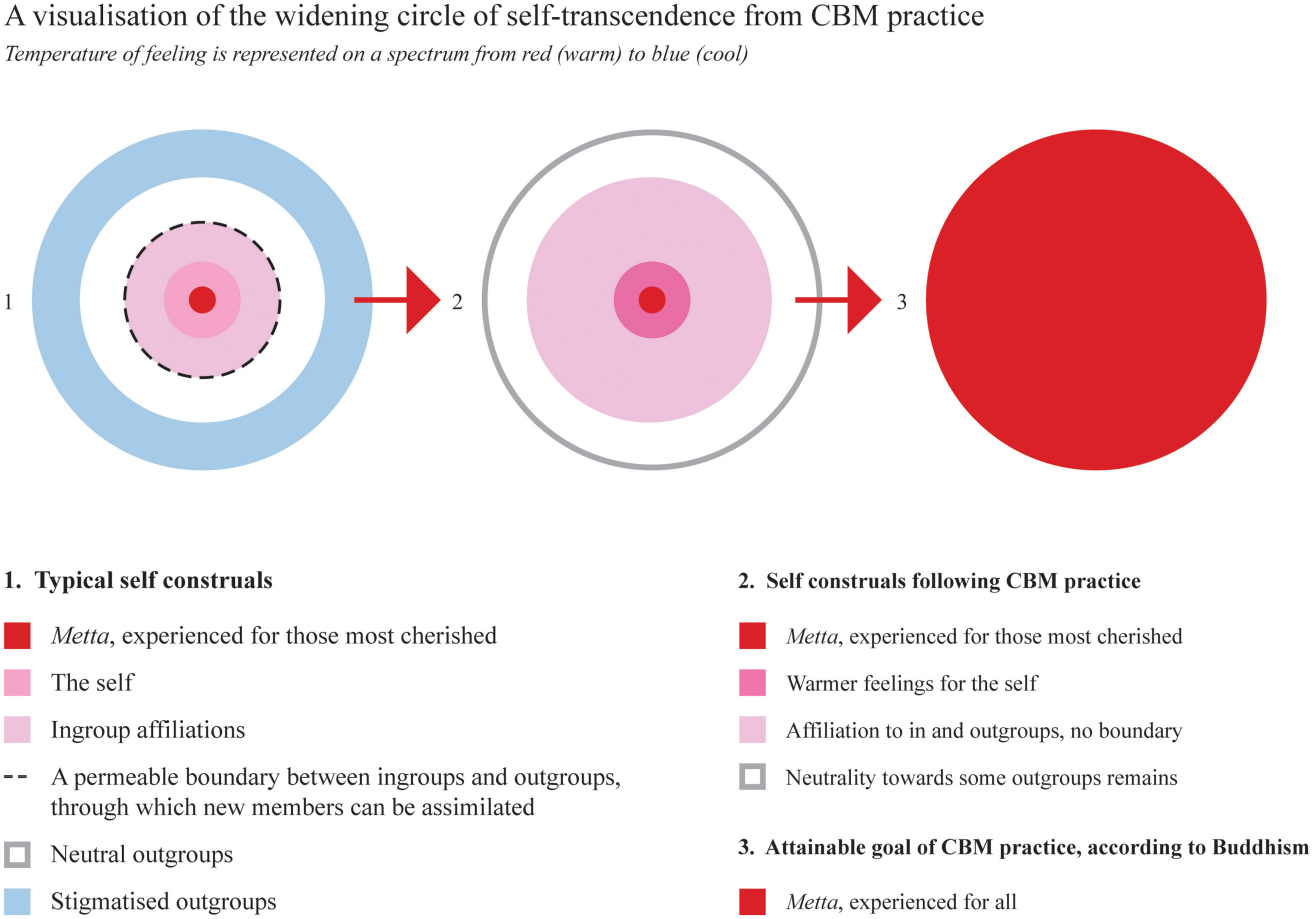
The widening circle of self-transcendence from compassion-based meditation (CBM) practice.

Self-transcendence, a shift in focus from self to other, is evidenced in feedback from participant #15, who expressed that it is “just so essential to view others as individuals and recognize that you are not the main character.” Participant #4 said that “it's easy to see how we are all part of one whole.” Similarly, participant #6 reported: “I learned to consider other people's wellbeing, and as someone that is naturally quite self-centered, I think this was an important lesson for me.” CBM helped participant #3 “be more present and conscious about how I arrive for other people.” This self-transcendence process is visualized in Figure 1, with the widening circle of *mettā* expanding self-construals and enhancing compassionate relationships with both the self and others.

## Discussion

Compassion-based meditation (CBM) focuses on the transformation of self-awareness through self-transcendence, which is particularly relevant in the context of structural inequalities in post-apartheid South Africa. This mixed methods pilot study was conducted to examine whether CBM practice could improve not only prejudicial, but also prosocial, intergroup outcomes in the South African context characterized by enduring historical racial conflict and power asymmetry. Preliminary results suggest that the sample of White adults who participated experienced greater subjective wellbeing and expressed more positive intergroup attitudes and prosocial tendencies following 8 weeks of CBM practice. Qualitative findings provided converging support, echoing CBM research in other settings associated with enhanced compassion for the self and others (Hofmann et al., 2011; Kang, 2019; Weng et al., 2013).

Post intervention, we observed significant increases in life satisfaction and reductions in perceived stress, and increasingly so, the greater CBM practice time participants reported. Positive intrapersonal effects described by participants included feelings of calm, positivity, increased presence, focus, awareness, groundedness, and tolerance of negative emotions. Reductions in irritability, anxiety, overthinking, reactivity, judgment, animosity, and anger were also reported. Significant positive changes in intergroup outcomes following the intervention included reduced affective prejudice toward all social groups, increased intergroup contact with both racial outgroups, and greater prosocial outcomes, as measured by collective action support and petitions supporting government restitution policies. It should be noted that all participants did not have the same experience throughout the 8-week CBM intervention, as some engaged in considerably more self-practice than others. Indeed, correlation analysis showed that those participants who engaged in more CBM self-practice sessions not only reported enhanced personal wellbeing, but also expressed greater outgroup compassion and reduced desire to socially distance from other racial groups. The qualitative findings suggest that participants made spontaneous connections between their increased wellbeing and improvements in their social experiences, reporting enhanced interpersonal peace, happiness, kindness, benevolent engagement, and a more positive view of all others. Their reports described a renewed awareness of others, eliciting feelings of empathy and openness, in many cases followed by a bridging of social distance in their daily lives.

A key finding emerging from participants' feedback describes a shift from self-orientation to greater affiliation with others, in line with Parks, Birtel and Crisp (2014) self-transcendence theory and the *Ubuntu* principle. Participants were guided to cultivate *mettā* through contemplating their most cherished relationships, sequentially extending this quality to themselves and their circles of affiliation, followed by those outside of relational circles. As the circle was broadened, conceivably inviting the expanding of self-construals to incorporate previous outgroup circles, the qualities of *mettā* can be seen to have extended further, stimulating the positive findings seen. With continued practice, the boundary between self and not-self is said to ultimately dissolve (see Figure 1), in accordance with Gilbert's (2005, p. 106) explanation of the change being “from a self-preservative to a species-preservative mode.” In effect, we could understand *mettā* as eventually subsuming all “others” into selfhood, where selfhood becomes synonymous with subjectively experienced oneness. This effect converges with *Ubuntu's* forgoing of individual objectives in service of collective wellbeing, suggesting that CBM may offer a culturally consonant vehicle for promoting intergroup affiliation in South Africa, with further study needed to assess its action in other social groups.

Participants commented on finding the workshop environment safe and non-judgemental. The function of the workshop sessions could be conceptualized as initiating and supporting the compassion practice, with those attending more workshop sessions also engaging in more self-practice between sessions. Importantly, enjoyment of other participants during the workshop sessions was not significantly associated with any positive outcomes, suggesting that the “active” ingredient was the compassion training itself. Many participants expressed the intention to continue their CBM practice going forward, motivated by the benefits already experienced. Participant 7 expressed: “I am now able to sit and offer compassion to myself and others, [and] notice periods of time or situations where a mindfulness practice will be useful or needed.”

This study's main objective reflects in feedback such as this by participant 6: “I specifically remember a time in the park, where I listened to … a part of the practice recording that asked me to imagine somebody in my mind. Near the park is a traffic guard, who always greets me when I walk past. That day I envisioned the man, properly picturing his face, his feelings and his wellbeing. Whilst before I had merely glanced at him.”

## Limitations and directions for future research

The small sample size prevents a generalization of the present findings to the broader population. We employed bootstrapping, FDR correction, and qualitative triangulation as strategies to mitigate this limitation, however, so that the results do provide an indication of trends that could reasonably be expected in a stronger powered study. In addition, while small sample sizes may inflate effect sizes, we reported Hedges' *g* effect sizes, which provide a correction factor to reduce positive bias when the sample size is small (Durlak, 2009). We observed large effects (>0.85) for subjective wellbeing and moderate effects for various intergroup outcomes, including prosocial behavior (0.44 <*g* < 0.75). These results are practically meaningful for practitioners and policymakers who seek effective interventions to promote complex social change.

The inclusion of an active control condition would be needed to distinguish conclusively the specific contribution of CBM practice from (i) experimental factors such as expectancy effects and social desirability bias, (ii) situational factors, such as external sociopolitical influences, and (iii) more generally from increased awareness or greater mindfulness (Aggarwal and Ranganathan, 2019; Van Dam et al., 2018). Active control conditions might include relaxation training, general discussion groups, mindful movement, or sham meditation. The absence of a control group currently limits any causal interpretations.

A factor in the present study that potentially obscured the extent of intergroup benefits possible, is the sample's fairly progressive intergroup attitudes pre-intervention. Because of high baseline attitudes, the scope of positive change may have been reduced in our sample, and the absence of significant results across some outcome variables, such as compassion for racial outgroups, could be understood in this light. These effects may have been exacerbated by self-selection bias, which can be reduced in future research by recruiting a more representative sample through stratified or purposeful sampling. Partnering with community leaders, tailoring recruitment material with culturally inclusive language, and removing structural barriers to participation could also assist in securing a more diverse sample (Watson-Singleton et al., 2019).

Despite these limitations, our findings suggest that CBM may be an area worthy of further exploration not only in the South African context, but also in other post-conflict or divided societies (e.g., Rwanda, Sri Lanka, Bosnia) to support the recovery from collective trauma. Conducting a larger randomized controlled trial (RCT) in future research would be necessary to validate the present statistical conclusions, while longitudinal studies would need to ascertain the long-term effects of CBM on intergroup relations and prosocial attitudes. The intervention should also be extended to other social groups (race and socioeconomic status), including people of color (Manuel, 2015). Transferring the methodology to other groups would need to be undertaken with an awareness of the added cultural and contextual complexities at play, however. CBM interventions have primarily been studied in western populations and may not be a good fit for all cultural groups. Additional piloting and conducting focus groups, for example, may be necessary to ascertain whether the present methodology is appropriate.

Finally, effective scaling of CBM interventions to address social divisions might be achieved through participatory adaptations, such as training community leaders as local facilitators of CBM practices (e.g., teachers, religious leaders, social workers), and integrating brief compassion practices into existing curricula (e.g., wellness, parenting, education) using locally resonant language and symbols (Potvin et al., 2023). In this way, age-appropriate and flexible CBM delivery options could be implemented in community, education, and religious settings, supported through cross-sectoral collaboration.

## Conclusion

Many of the benefits associated with CBM practice in other settings were seen to translate to a South African context in this pilot study, offering promising initial support for the potential of CBM to enhance positive outgroup attitudes and boost prosocial outcomes in South African adults. In contexts of high prejudice where other social interventions driving structural reform may have stalled, dismantling the barriers to connection may well be an “inside job.”

## Data Availability

The raw data supporting the conclusions of this article will be made available by the authors, without undue reservation.

## Footnotes

^1^We acknowledge that these racial constructions are not based in biological realities or markers of inherent identities. Rather, racial identifiers are carriers of social and political meaning, in flux over time and space (Dolby, 2001). It is in this spirit that race labels are used in the present study, with an acknowledgment of the heterogeneities that they represent and the violence inherent in these terms.

^2^The term “other” is used to signify another with whom the subject does not identify, i.e., an outgroup member, conceptually mapped outside of self-construals.

^3^*Positionality Statement:* Mindful that social identities can influence one's approach to research and interpretation of data (Roberts et al., 2020), the authors wish to provide the reader with potentially relevant information about their backgrounds. With respect to race and nationality: all authors identify as White and South African. While conscious of the intersectional complexity and heterogeneity within socially constructed categories, we believe referring to Black African, Colored, and White people is warranted, without intending to oversimplify, given the legacies of systematic racial discrimination under colonialism and apartheid in South Africa.
