# Supplementary material for: A kind mind: effects of compassion-based meditation on prosocial intergroup outcomes in a South African pilot sample
Source: Front Psychol. 2025 Jul 11;16:1450549. doi: 10.3389/fpsyg.2025.1450549 (PMC12291553; doi:10.3389/fpsyg.2025.1450549)
Supplement: Supplementary file 1 [file Data_Sheet_1.docx]

**A kind mind: Effects of compassion-based meditation on prosocial
intergroup outcomes in a South African pilot sample**

# Supplementary Material

# Survey sections

Survey headings are provided for ease of reference, but were not included in the pre- and post-test surveys.

*Satisfaction with Life Scale*

Below are five statements that you may agree or disagree with. Indicate your agreement with each item using the scale below. Please be open and honest in your responding.

**1 2 3 4 5 6 7**

*strongly neither agree strongly*

*disagree nor disagree agree*

1. In most ways my life is close to my ideal.
2. The conditions of my life are excellent.
3. I am satisfied with my life.
4. So far I have gotten the important things I want in life.
5. If I could live my life over, I would change almost nothing.

*Perceived Stress Scale*

The questions below ask about your feelings and thoughts during the last two weeks. In each case, you will be asked to indicate how often you felt or thought a certain way.

**0 1 2 3 4**

*never almost never sometimes fairly often very often*

In the last two weeks:

1. How often have you been upset because of something that happened unexpectedly?
2. How often have you felt that you were unable to control the important things in your life?
3. How often have you felt nervous and “stressed”?
4. How often have you felt confident about your ability to handle your personal problems?
5. How often have you felt that things were going your way?
6. How often have you found that you could not cope with all the things that you had to do?
7. How often have you been able to control irritations in your life?
8. How often have you felt that you were on top of things?
9. How often have you been angered because of things that were outside of your control?
10. How often have you felt difficulties were piling up so high that you could not overcome them?

*Feeling thermometers*

Please indicate below how cold (unfavourable) or warm (favourable) you feel towards each of the following groups:

**0** *very cold very warm* **100**

1. Thermometer rating for Black African people
2. Thermometer rating for Coloured people
3. Thermometer rating for White people

*Social Distance*

Please rate your agreement with these statements using the sliders:

**0** *strongly disagree*   *strongly agree*  **100**

1. It would bother me if my son or daughter ended up marrying a Black African person.
2. It would bother me if my son or daughter ended up marrying a Coloured person.
3. It would bother me if I had to live in the same house/flat as a Black African person.
4. It would bother me if I had to live in the same house/flat as a Coloured person.
5. I would not want my child to be in a school where most of the other children are Black African.
6. I would not want my child to be in a school where most of the other children are Coloured.

*Intergroup contact*

From the options provided, please answer the following questions about your interactions with Black African and Coloured people. Do not think too long about the answers, rather give the first answer that comes to mind.

1. In general, how often do you interact with [Black African/Coloured] people?

**1 2 3 4 5 6 7**

*never rarely sometimes quite a bit often very often all the time*

2. When you do interact with [Black African/Coloured] people, how often are the interactions positive/pleasant versus negative/unpleasant?

**1 2 3 4 5 6 7**

*always as often negative always*

*negative as positive positive*

*Outgroup compassion*

Please indicate how sorry you feel for Black African/Coloured/White people in the following situations:

**0** *not sorry for them at all*  *very sorry for them* **100**

Scenarios, Set 1:

1. Black African people whose children have a hard time at school.
2. Black African teenagers who get pregnant accidentally.
3. Black African adults who receive a very low salary.

Scenarios, Set 2:

1. White people whose children are bullied at school.
2. White teenagers who become addicted to drugs.
3. White adults who are not able to find a job.

Scenarios, Set 3:

1. Coloured people whose children don't have friends at school.
2. Coloured teenagers who become infected with HIV/Aids.
3. Coloured people who struggle to make ends meet.

*Collective action support*

How much do you support each of the following?

**0** *strongly oppose*   *strongly support*  **100**

1. Poor students protesting in favour of reduced fees and/or free tertiary education.
2. Workers and trade union members protesting on behalf of people outside of formal employment.
3. Communities from informal settlements protesting over slow land reform.
4. Impoverished communities engaging in service delivery protests for basic municipal services.

*Petition Support*

Below are some petitions concerning a number of relevant social issues that have been distributed online in South Africa. Please let us know if you would like your vote counted for (or against) each of these petitions:

|  | Add my user ID **to support** the petition | Do not add my user ID to the petition | Add my user ID **in opposition** to the petition |
| --- | --- | --- | --- |
| A wealth tax on the 10% richest individuals. |  |  |  |
| Broad-based Black Economic Empowerment  (B-BBEE) |  |  |  |
| Free tertiary education for Black students who cannot afford tuition fees. |  |  |  |
| Affirmative Action and employment equity targets  in government and private organisations. |  |  |  |
| The controlled expropriation of White-owned land without compensation. |  |  |  |

*Compassion-based meditation (CBM) practice* (post-test)

1. How many sessions did you attend or watch the recording of, out of the 8 weeks?
2. How many self-practice sessions did you complete in the period between the first and last workshop sessions?
3. On adding up your total self-practice time, how much time did you spend meditating in this period (in minutes)? *[coded 1–20]*
4. How much did you enjoy the other participants in the group sessions? [0 *= not at all;* 100 *= very much]*

*Open-ended (qualitative) questions* (post-test)

1. Please share any experiences that stand out for you of the workshop, positive or negative.
2. Please share something important that you learned and what value it had for you with regard to your interactions with other people.
3. Do you have suggestions for what could have made your experience more rewarding?

**Compassion Meditation**

The 8 weekly full compassion meditation practices can be accessed using the following links:

<https://insighttimer.com/michalgeorge/guided-meditations/circle-of-kindness-part-1>

<https://insighttimer.com/michalgeorge/guided-meditations/circle-of-kindness-part-2>

<https://insighttimer.com/michalgeorge/guided-meditations/circle-of-kindness-part-3>

<https://insighttimer.com/michalgeorge/guided-meditations/circle-of-kindness-part-4>

<https://insighttimer.com/michalgeorge/guided-meditations/circle-of-kindness-part-5>

<https://insighttimer.com/michalgeorge/guided-meditations/circle-of-kindness-part-6>

<https://insighttimer.com/michalgeorge/guided-meditations/circle-of-kindness-part-7>

<https://insighttimer.com/michalgeorge/guided-meditations/circle-of-kindness-part-8>
